# Acceptability of the Vaccine against COVID-19 in Spanish Health Sciences Students: A Cross-Sectional Study

**DOI:** 10.3390/ijerph191912244

**Published:** 2022-09-27

**Authors:** Noelia Rodríguez-Blanco, Nancy Vicente-Alcalde, Laura Cubero-Plazas, Jesús Sánchez-Más, Emilia Montagud, Raul Moragues, Eva Gabaldón-Bravo, Jose Antonio Hurtado-Sanchez, José Tuells

**Affiliations:** 1 Nursing and Physical Therapy Department, Health Sciences Faculty, CEU-Cardenal Herrera University, CEU Universities, Plaza Reyes Católicos, 19, 03204 Elche, Spain; 2 Department of Obstetrics and Gynaecology, Marina Baixa University Hospital, Av. Alcalde En Jaume Botella Mayor, 7, 03570 Villajoyosa, Spain; 3 Penitentiary Center Alicante II, General Secretariat of Penitentiary Institutions, 03400 Villena, Spain; 4 Departament of Nursing, Faculty of Medicine and Health Sciences, Catholic University of Valencia San Vicente Mártir, Espartero 7, 46007 Valencia, Spain; 5 Biomedical Sciences Department, Health Sciences Faculty, CEU-Cardenal Herrera University, CEU Universities, Plaza Reyes Católicos, 19, 03204 Elche, Spain; 6 Servicio de Farmacia de Atención Primaria, Departamento de Salud de Torrevieja, 03186 Torrevieja, Spain; 7 Faculty of Health Sciences, Universidad Católica de San Antonio, 30107 Murcia, Spain; 8 Center of Operations Research (CIO), University Miguel Hernandez of Elche (UMH), 03202 Elche, Spain; 9 Department of Nursing, Faculty of Health Sciences, University of Alicante, 03690 San Vicente del Raspeig, Spain; 10 Department of Community Nursing, Preventive Medicine and Public Health and History of Science, University of Alicante, 03690 San Vicente del Raspeig, Spain

**Keywords:** COVID-19, vaccine, acceptability, students, health sciences, nursing, pharmacy, medicine

## Abstract

Healthcare professionals must play an exemplary role in the field of vaccinology. It is convenient that they are trained during their time at university. The objective of this study was to determine the acceptability of the vaccines against COVID-19 in health sciences students in Spanish universities. A cross-sectional study was performed regarding the acceptance of the vaccines against COVID-19 in students in the Health Sciences Degrees in Spanish universities was performed on a sample of students of nursing, medicine, and pharmacy during the spring of 2021, via an online questionnaire with 36 questions designed ad hoc, self-administered, anonymized, and standardized. There were 1222 students participating, of Spanish nationality (97.4%), women (80.5%) and with an average age of 22.0 ± 4.8 years old. Of those, 12.3% had had the disease, 44.0% had to quarantine, 70.8% had undergone diagnostic tests, out of which 14.1% were positive. In total, 97.5% of those surveyed indicated their desire of being vaccinated, if possible, with Comirnaty^®^ (74.9%). At the time of the study, 49.6% were already vaccinated. The reasons for vaccination differed according to the degree and the doubts about vaccine safety was the largest reason for reluctance. Some 37.7% suspected that there are unknown adverse effects and 85.6% of those vaccinated experienced some mild effects after injection. Vaccine acceptance and confidence in the recommendations given by health authorities is high in health sciences students.

## 1. Introduction

Vaccination has shown its efficacy as a public health tool to prevent various infectious diseases through the last two centuries, a fact which has been ratified during the current COVID-19 pandemic [1]. In this case, the vaccine deployment has been a confirmed hope to mitigate it, even though the mass vaccination strategy has been unequal and not equitable [2]. The safety of vaccines has been as much, if not more, a concern than their own efficacy, so that the WHO and scientific societies have repeatedly affirmed that vaccines currently have an excellent safety history [3].

Having a vaccine is not equivalent to its immediate acceptance, an effect described as “Pandemic Public Health Paradox”, which associates the acceptance of a vaccine to the media impact rather than the epidemiological dynamics [4]. There are several factors that can be an obstacle to achieving acceptance of a vaccine, such as: geographic location, logistics, health system, accessibility, availability, knowledge of affordable vaccines, as well as psychological, cognitive, emotional, cultural, social factors. and politicians. The latter has recently been described in the US with vaccination for COVID-19, where counties with a high percentage of Republican voters had significantly lower vaccination rates [5,6].

Another recent study on COVID-19 vaccine uptake in 13,426 randomly selected people from 19 countries found that a transparent, evidence-based policy was needed to prevent refusal of new vaccines [7]. People who were not concerned about infection (57%; *p* ≤ 0.001) had the highest rates of COVID-19 vaccine reluctance [8].

In the case of the vaccination against COVID-19, a very present threat has been caused by “infodemia”, the “disinformation” and “misinformation”, a very visible fact in this occasion, but which is not new in the history of vaccines [9,10,11]. At a global level, the population has been following the process of the vaccines against COVID-19, it has asked the healthcare professionals and has informed itself via the media, in a search for reassuring information which minimizes their possible doubts or rejections [12,13,14]. Reducing the concerns about the secondary effects of these new vaccines is achieved by providing correct and truthful information [15,16,17].

The determinant role of healthcare professionals in achieving higher vaccination coverage has been noted, as well as their role as a main source of information, the most influential one, for the general population [18,19,20]. This brings to the fore the importance of the training of the students of health sciences in the field of vaccines, not only by providing them with the specific knowledge, but also by modelling their positive attitude towards vaccination [21,22].

Healthcare professionals are the main recruiters for the vaccination process, since they use their time to advise patients, parents, families and the public about the benefits, risks, and safety of vaccines, as well as their route of administration [23,24,25]. They are also in charge of detecting and notifying to the health authorities the possible side effects of the vaccines [26]. The evidence shows that educational interventions are the best strategy to improve the adherence, attitude, and knowledge of the students about vaccines, since a multidisciplinary orientation of teaching contributes to going beyond the traditional approach of health education [23].

In this context, including the first line healthcare and socio-health workers as priority groups to vaccinate in the Vaccination Strategy of the National Healthcare System (SNS), was an exemplary act towards the acceptance of the vaccine by the population [27].

It is in the hands of the healthcare workers to lead the changes towards the improvement of the health of the population, and these must start from the health sciences classrooms [28]. The goal of this study was to determine the acceptance of vaccines against COVID-19 in students of university degrees in health sciences (Nursing, Medicine and Pharmacy) in various Spanish universities.

## 2. Materials and Methods

### 2.1. Design and Participants

A cross-sectional study was performed regarding the acceptance of the vaccines against COVID-19 in students in the Health Sciences Degrees in Spanish universities. The inclusion criteria were being a student of the Degrees in Nursing, Medicine, or Pharmacy in any of the participating Spanish universities. The criteria for exclusion were refusal to participate in the study or answering to the open-ended questions inappropriately.

### 2.2. Tool

The tool used was an online questionnaire via the Google Docs application, self-administered, anonymous and standardized, to evaluate the acceptance of the COVID-19 vaccine in students of the Degrees in Nursing, Medicine, and Pharmacy in Spanish universities. A pilot study was carried out with a group of 25 nursing students, who were not considered for this analysis. This pilot study allowed us to collect the impressions of the respondents, which were then evaluated by a group of experts to assess the understanding of the questions by the respondents and to determine the time it took them to complete the questionnaire and check the operation. internal. consistency of all the components of the questionnaire. The final questionnaire, designed ad hoc for this study, was made up of a total of 36 questions, some of which were multiple choice and others with a nominal scale. It consisted of four sections: (i) basal sociodemographic questionnaire (sex, age, origin, civil state, academic year), and personal health variables (items 1 to 15: suffering a chronic disease, use of tobacco, alcohol or psychoactive drugs); (ii) COVID-19 variables (items 11 to 16: having had COVID-19, contagion in the university environment, need for quarantine, death of some family member or friend due to COVID-19, PCR test, PCR results); (iii) acceptance of the COVID-19 vaccine (items 17 to 36: vaccination status, possibility of choice of vaccine, complete infant vaccination, previous flu vaccination, adverse effects of the vaccine, trust in the health authority recommendations, reasons for acceptance or rejection of the vaccine).

The questionnaire was designed after a literature review, with the last version approved by consensus of the team of researchers.

### 2.3. Procedure

The sample was composed of students who responded to the self-administered survey carried out during the months of May and June 2021. To use an appropriate sampling frame, it was decided to recruit the participants via the decanal team in their universities of origin, who provided them with information about the study, the call for participation, the informed consent, and the survey via their university email account.

Every person who showed willingness to participate and met the criteria for inclusion was included. To foment participation, the decanal team of each university sent, via email and multiple times, the details of the study, encouraging participation. The sample was by convenience on a non-probabilistic bias.

The mean ± standard deviation was utilized for the quantitative variables, and frequency tables were used for the qualitative data. The Chi-square test was utilized to investigate the relationships between the categorical variables. The factors associated to the willingness to receive the vaccine were identified through the use a logistic regression analysis. A multivariate logistic regression was performed to identify the adverse effects associated with the type of vaccine received, with the odds ratio (OR) probability and a confidence interval (CI) of 95% calculated. The likelihood was calculated with Wald Chi-square test, and the goodness-of-fit was tested by Pearson’s test. The statistical analysis was performed with the IBM SPSS Statistics for Windows, version 24.0 (SPSS Inc., Chicago, IL, USA).

### 2.4. Ethical Considerations

The study was approved by the Ethics Committee of the University of Alicante. Participation in the survey was anonymous and voluntary, and the questionnaire was treated completely confidentially, ensuring the privacy of the data of those surveyed. This study was conducted according to the ethical guidelines for medical research in human beings established in the Declaration of Helsinki and the EU Regulation 134 2016/679 (GDPR) relating to the treatment of personal data.

## 3. Results

### 3.1. Population Description

A total of 1222 students of health sciences subjects took part in the survey, with most of them being of Spanish nationality (97.4%), female (80.5%), living alone (67.3%) and with an average age of 22.0 ± 4.8 years. Of them, 76.5% (936) of the students were from the following universities: Catholic University of Valencia (UCV) 29.3% (359), University of Alicante (UA) 20.5% (251), Saint Anthony Catholic University of Murcia (UCAM) 17% (208) and University of Jaen (UJA) 9.6% (118).

As shown in Table 1, the participation oscillated between 37.2% and 62.1% among academic years and degrees. Most participants reported that they do not suffer any chronic disease (89.0%) and that they do not use tobacco (87.0%), alcohol (89.6%) or psychoactive drugs (98.3%). Among the university students, those studying nursing reported higher percentage of tobacco use (*p* = 0.045) and higher presence of chronic diseases (*p* = 0.015). There were no significant differences regarding the consumption of alcohol (*p* = 0.07) or psychoactive drugs (*p* = 0.767) among degrees.

### 3.2. Variables Relating to the COVID-19 Disease

Of the participating students, 12.3% (n = 150) had had COVID-19, most of which (81.3%) claim that they were not infected at the university. 44.3% (n = 534) had to be quarantined due to close contact with some case and 16.6% (n = 203) reported the death of some family member or friend due to COVID-19. There were no significant differences between the degrees for these variables.

Most of the students had to have some diagnostics test performed on them: PCR or antigen test (70.8%). The degrees of Nursing and Medicine were those with a higher percentage of students having taken PCR or antigen tests (*p* < 0.001). In any case, the vast majority tested negative (Table 2)

### 3.3. Variables Relating to the Acceptance of Vaccines in General and against COVID-19

As shown in Table 3, most of the students of all three degrees would vaccinate against COVID-19 and, out of these, 58.5% believe that there should be a choice of which vaccine to have, with the Comirnaty^®^ vaccine being the most frequently cited option. At the time of the survey, half the students had already received the vaccine, with Comirnaty^®^ being the most administered among nursing and medicine students (91.9%), while among those studying pharmacy it was Vaxzevria^®^ (91.7%). In Spain you cannot choose the type of vaccine, they are recommendations from health institutions by risk group and availability of vaccines at that time (27).

Regarding acceptance of vaccines in general, 97.5% of those surveyed claimed having a complete childhood vaccination schedule, even though only 59.8% claimed to have ever had a flu vaccination. We found significant differences among the different health degrees. The pharmacy students reported a smaller percentage having a complete childhood vaccination schedule, as well as a lower acceptance of the flu vaccine compared to the nursing or medicine students (*p* < 0.001).

### 3.4. Justified Reasons for the Acceptance or Rejection of the Vaccines against COVID-19

**Figure 1:**
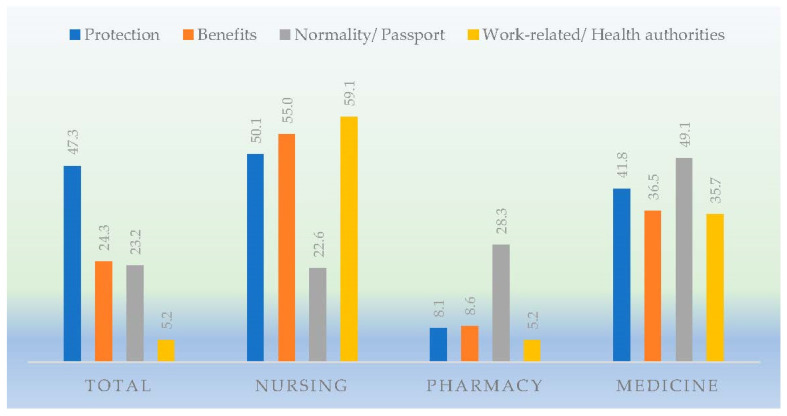
Reasons for the acceptance towards the vaccines against the COVID-19 disease in students of the three degrees.

**Figure 2:**
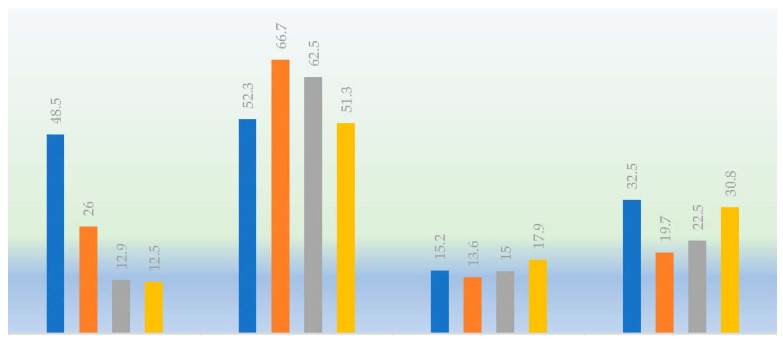
Reasons for the rejection towards the vaccines against the COVID-19 disease in students of the three degrees.

The reasons for the acceptance or rejection towards the COVID-19 vaccine are shown in Figure 1 and Figure 2. The reasons for the acceptance of the vaccines are associated with an attitude of individual and community protection from the disease. The most frequently cited reasons by the majority of the students were 1. Protection of others and myself. 2. The benefits of vaccines. 3. Going back to normality and obtaining the immunization passport. 4. Work-related reasons, face-to-face placements, and on the recommendation of the authorities.

The pharmacy degree showed a lower proportion of students who preferred these reasons with respect to the degrees in nursing or medicine (*p* < 0.001). Among the degrees, it is worth highlighting that the main reason for acceptance of the vaccine in the pharmacy degree was the normality/passport, whereas it was the least determinant reason for the nursing and medicine students.

The work-related reasons or the obligation to be vaccinated to carry out the clinical placements of the degree or the health recommendation were the reasons that had the least incidence towards acceptance of the vaccine among the health students, particularly among the pharmacy students.

In Figure 2, we can see the reasons for the rejection towards the vaccines against COVID-19. The doubts about vaccine safety or the lack of knowledge about the disease were the most cited reasons.

However, and even though it was cited less often, none of the three collectives considered themselves groups at risk, and they did not even see this vaccine as necessary, despite being future healthcare workers.

### 3.5. Variables Relating to the Secondary Effects of the Vaccines against COVID-19

Even though most students do not believe that the COVID-19 vaccine has effects more serious than the rest of vaccines in the vaccination schedule, 37.7% (n = 461) of the participants in the study believe that the approved vaccines against COVID-19 have unknown side effects. The students in the pharmacy degree are those who cite these effects the most (*p* = 0.026) (Table 4).

Most students trust the vaccination recommendations against COVID-19 made by the health authorities (89.2%); however, the number of pharmacy students who trust them is lower (82.1%) than among the nursing students (91.2%) or medicine (87.7%) (*p* = 0.035). In total, 10.4% acknowledged a lack of confidence.

When asked about the adverse effects after vaccination, most of those surveyed reported having suffered adverse effects (85.6%), without significant differences among the health degrees. The most prevalent adverse effects were pain at the injection site and tiredness. The pharmacy students showed significantly higher occurrence of fever (0 = 0.018) and shivering (*p* = 0.031).

### 3.6. Multivariate Logistic Regression between the Administered Vaccines and the Declared Adverse Effects

Table 5 shows the proportion of health students who suffered the certain adverse effects surveyed in relation to the type of vaccine administered.

Receiving the Vaxzevria^®^ vaccine revealed a higher percentage of appearance of all evaluated adverse effects with respect to the administration of Comirnaty^®^ or Spikevax^®^, except for the occurrence of pain at the injection site and swollen glands, which showed no differences among the three administered vaccines. The multivariate logistic regression analysis confirms that the type of vaccine administered is an influential factor in the occurrence of certain adverse effects. Thus, when comparing with Comirnaty^®^, the administration of the Vaxzevria^®^ vaccine was associated with a higher number of cases of fever (OR = 0.18, *p* < 0.001), shivering (OR = 0.58, *p* = 0.044), and vomiting (OR = 0.09, *p* = 0.003). When compared with Spikevax^®^, the administration of the Vaxzevria^®^ vaccine was associated with a higher number of cases of fever (OR = 0.27, *p* < 0.001) and shivering (OR = 0.29, *p* = 0.003), but lower pain at the injection site (OR = 2.4, *p* = 0.026).

## 4. Discussion

This study has been able to identify the degree of acceptance of the vaccines against COVID-19 in Spanish health sciences students, who have indicated a very high intention to be vaccinated (97.5%), a result higher than other previous studies in students in USA (52.5%), France (58.0%) or China (78.9%) [29,30,31].

This collective is of special relevance since during their careers they will play a role of vaccination assessment and recommendation to the population, as reflected by various studies [32,33,34,35,36,37,38,39,40,41,42,43,44,45].

This research was carried out during the spring of 2021, a period between the fourth and the fifth pandemic waves in Spain, with the students completing their clinical placements in hospitals or healthcare facilities and almost half of them already being vaccinated (49.6%), especially those studying nursing (60.5%). Since the beginning of the pandemic, almost half of the students had been quarantined due to close contact or infection. Moreover, they had undergone diagnostic tests, especially in the disciplines of nursing and medicine. Those who had experienced the disease (12.3%) did not refer to the university as their location of infection.

The surveyed students trust in vaccines as a preventive method and consider it important to have a complete vaccination schedule. However, only slightly more than half reported having been vaccinated at any point against the flu, with the pharmacy students being those who had been vaccinated significantly less, a result like those of other studies [39,46].

The main reason towards acceptance of vaccination was found to be the protective effect that vaccines have on the community and themselves [47], followed by the known benefits of vaccinations and the need to return to a state of normality. Pharmacy students highlighted obtaining the COVID passport and work reasons as reasons; however, nursing and medical students who had closer contact with the disease due to their care work, indicated protection as the main reason. The acceptability of the vaccine was high in all three disciplines, even though it was higher among the medicine students, followed by those of nursing [48]. In 2021, Nguyen [49] highlighted the necessity for vaccination against COVID in healthcare staff, so it is striking that most of the surveyed students do not consider themselves a group at risk, as future healthcare workers.

Vaccine hesitancy is expressed in relation to the safety and efficacy of the vaccines against COVID-19 and the lack of knowledge about the disease itself, which agrees with previous studies [24,30,39]. Over a quarter of the students believe that these new vaccines can have unknown adverse effects, a common reason when new vaccines are added to the vaccination schedule and observed among healthcare professionals [41]. Other factors contributing to vaccine hesitancy include concerns about the secondary effects and lack of trust in the information received from public health experts [29].

The proportion of adverse effects depending on the type of COVID-19 vaccine received confirms that the administered vaccine is an influential factor in the occurrence of certain adverse effects. Most students believed that it should be possible to choose which vaccine to have administered, with the favorite one being Comirnaty^®^. The type of vaccine administered is an influential factor in the occurrence of certain adverse effects, as can be appreciated when comparing those from the Comirnaty^®^ vaccine with the Vaxzevria^®^ vaccine, with there there is statistically significantly more fever, shivering, and vomiting associated to the latter one. When Spikevax^®^ was compared with Vaxzevria^®^, this one also caused a higher number of cases of fever and shivering, but less pain at the injection site. In general, all adverse effects were of a mild nature (pain at injection site, muscular pain, tiredness, or fever).

Regarding the healthcare authority issued recommendations, those surveyed manifested a good acceptance, although this statement can be improved, since 10.4% would not accept these recommendations. Despite this, this study shows better results than that of Mascarenhas AK et al. [50], where only 65.6% of the odontology students trusted the recommendations issued by the public health experts. The professionals who showed more confidence in the information provided by the health authorities are those who are more likely to vaccinate, as informed by the work by Kelekar AK et al. [42].

Even though the vaccines against COVID-19 are new, and can thus generate greater concern, the morbidity and mortality results observed during the pandemic may encourage professionals to get vaccinated despite their concerns [44]. Recently, the necessity of paying special attention to the inclusion in the study plans of topics which explain vaccines in all their processes has been highlighted [30,40,41], a view we fully share.

The main practical implication of this study is the verification of the need to train future health professionals on the potential benefits of an adequate vaccination of citizens. Therefore, the specific contents on vaccines that must be included in the training of future health professionals must be related to vaccine safety and its composition, in addition to continuous training during their professional activity due to the constant changes that require updating and recycling. Higher levels of confidence were positively related to the intention to accept vaccination. Confidence in governments improves the level of acceptance of the recommendations given [51].

The main limitations of our study were that a validated questionnaire was not found, which is not available in the scientific literature because COVID-19 is an emerging disease and the number of participants in the three degrees is not similar. However, the large sample obtained from professionals who will be the main advisors on vaccination of the population is an important strength.

## 5. Conclusions

The SARS-CoV-2 pandemic has highlighted the importance of vaccination and the role of healthcare professionals in their implementation. Health sciences students represent the future in maintaining a high level of vaccine acceptance. They will be seen by the population as the most reliable source of information. The high level of confidence that students show for the recommendations given by the health authorities is also a positive predictor. Therefore, the results of this study are encouraging, and support the promotion of active teaching of vaccines during the training period and further exploration of research in this area.

## Figures and Tables

**Table 1:** Demographic characteristics of college students (n = 1222).

	Nursing n (%)	Pharmacy n (%)	Medicine n (%)	Total n (%)	IC 95%	*p*-Value
	716 (52.0)	124 (11.5)	382 (36.5)	1222 (100)		
Sex						
Male	95 (13.3)	46 (37.1)	97 (25.4)	238 (19.5)	(14.5–24.5)	<0.001
Female	621 (86.7)	78 (62.9)	285 (74.6)	984 (80.5)	(78.0–82.9)	
Origin						
Spanish	697 (97.3)	120 (96.8)	373 (97.6)	1190 (97.4)	(96.5–98.3)	0.867
Not Spanish	19 (2.7)	4 (3.2)	9 (2.4)	32 (2.6)	(0.0–8.1)	
Living arrangement						
Alone	487 (68.0)	69 (55.6)	266 (69.6)	822 (67.3)	(64.1–70.5)	0.013
With a partner	229 (32.0)	55 (44.4)	116 (30.4)	400 (32.7)	(28.1–37.3)	
Age (years)	22.2 ± 5.0	22.8 ± 5.2 *	21.6 ± 4.1 †	22.0 ± 4.8		
Academic year						
Lower	376 (52.5)	47 (37.9)	142 (37.2)	565 (46.2)	(42.1–50.3)	<0.001
Upper	340 (47.5)	77 (62.1)	240 (62.8)	657 (53.8)	(50.0–57.6)	
Chronic disease						
No	622 (86.9)	113 (91.1)	353 (92.4)	1088 (89.0)	(87.1–90.8)	0.015
Yes	94 (13.1)	11 (8.9)	29 (7.6)	134 (11.0)	(5.7–16.3)	
Tobacco						
No	609 (85.1)	109 (87.9)	345 (90.3)	1063 (87.0)	(84.9–89.0)	0.045
Yes	107 (14.9)	15 (12.1)	37 (9.7)	159 (13.0)	(7.7–18.2)	
Alcohol						
No	643 (89.8)	104 (83.9)	348 (91.1)	1095 (89.6)	(87.8–91.4)	0.070
Yes	73 (10.2)	20 (16.1)	34 (8.9)	127 (10.4)	(5.1–15.7)	
Psychoactive drugs						
No	705 (98.5)	121 (97.6)	375 (98.2)	1201 (98.3)	(97.6–99.0)	0.767
Yes	11 (1.5)	3 (2.4)	7 (1.8)	21 (1.7)	(0.0–7.2)	

* *p* = 0.035 vs Nursing; † *p* = 0.008 vs Pharmacy (Test U Mann-Whitney). The Chi2 *p*-value was calculated using contingency tables. Academic year: Lower: 1–2 course and Upper 3–5 course.

**Table 2:** COVID-19 disease in surveyed students. (n = 1222).

	Nursing n (%)	Pharmacy n (%)	Medicine n (%)	Total n (%)	IC 95%	*p*-Value
Have you had COVID-19? (n = 1222)						
Yes	100 (14.0)	15 (12.1)	35 (9.2)	150 (12.3)	(7.0–17.5)	0.069
No	616 (86.0)	109 (87.9)	347 (90.8)	1072 (87.7)	(85.7–89.6)	
Did you catch it at university? (n = 150)						
Yes	4 (4.0)	0 (0)	0 (0)	4 (2.7)	(0–18.6)	0.527
No	78 (78.0)	13 (86.7)	31 (88.6)	122 (81.3)	(74.4–88.2)	
Don’t know	18 (18.0)	2 (13.3)	4 (11.4)	24 (16.0)	(1.3–30.6)	
Have you had to quarantine? (n = 1213)						
Yes	316 (44.4)	50 (40.7)	168 (44.3)	534 (44.0)	(36.8–51.8)	0.729
No	395 (55.6)	73 (59.3)	211 (55.7)	679 (56.0)	(49.0–62.4)	
Have any of your family members or friends died from COVID-19? (n = 1222)						
Yes	118 (16.5)	17 (13.7)	68 (17.8)	203 (16.6)	(11.5–21.7)	0.562
No	598 (83.5)	107 (86.3)	314 (82.2)	1019 (83.4)	(81.1–85.7)	
Have you taken a diagnostic test, antigen test or PCR? (n = 1222)						
Yes	526 (73.5)	70 (56.5)	269 (70.4)	865 (70.8)	(67.7–73.8)	<0.001
No	190 (26.5)	54 (43.5)	113 (29.6)	357 (29.2)	(24.5–34.0)	
What were the results of the antigen test or PCR? (n = 865)						
Positive	77 (14.6)	13 (18.6)	32 (11.9)	122 (14.1)	(7.9–20.3)	0.307
Negative	449 (85.4)	57 (81.4)	237 (88.1)	743 (85.9)	(83.4–88.4)	

*p*-value = calculated for the Chi-square test group.

**Table 3:** Acceptance of the COVID-19 vaccine and attitude towards vaccination. (n = 1222).

	Nursing n (%)	Pharmacy n (%)	Medicine n (%)	Total n (%)	IC 95%	*p*-Value
Would you vaccinate against COVID-19? (n = 1222)						
Yes	695 (97.1)	120 (96.8)	377 (98.7)	1192 (97.5)	(96.6–98.4)	0.214
No	21 (2.9)	4 (3.2)	5 (1.3)	30 (2.5)	(0–8.0)	
Should there be a choice of COVID-19 vaccine? (n = 1222)						
Yes	434 (60.6)	73 (58.9)	208 (54.5)	715 (58.5)	(54.8–62.1)	0.142
No	282 (39.4)	51 (41.1)	174 (45.5)	507 (41.5)	(37.2–45.8)	
Which vaccine would you like to be given? (n = 1222)						
Comirnaty^®^	542 (75.7)	82 (66.1)	291 (76.2)	915 (74.9)	(72.1–77.7)	0.043
Spikevax^®^	57 (8.0)	13 (10.5)	41 (10.7)	111 (9.1)	(3.7–14.4)	
Vaxzevria^®^	77 (10.8)	15 (12.1)	29 (7.6)	121 (9.9)	(4.6–15.2)	
Jcovden^®^	40 (5.6)	14 (11.3)	21 (5.5)	75 (6.1)	(0.6–11.5)	
Have you received the COVID-19 vaccine? (n = 1222)						
Yes	433 (60.5)	24 (19.4)	149 (39.0)	606 (49.6)	(45.6–53.6)	<0.001
No	283 (39.5)	100 (80.6)	233 (61.0)	616 (50.4)	(46.5–54.3)	
If you have received it, which vaccine did you receive? * (n = 606)						
Comirnaty^®^	221 (51.0)	1 (4.2)	61 (40.9)	283 (46.7)	(40.9–52.5)	<0.001
Spikevax^®^	49 (11.3)	1 (4.2)	22 (14.7)	72 (11.8)	(4.4–19.2)	
Vaxzevria^®^	163 (37.6)	22 (91.7)	66 (44.3)	251 (41.4)	(35.63–47.5)	
Do you have a complete childhood vaccination schedule? (n = 1222)						
Yes	705 (98.5)	112 (90.3)	374 (97.9)	1191 (97.5)	(96.6–98.4)	<0.001
No	6 (0.8)	6 (4.8)	3 (0.8)	15 (1.2)	(0–6.7)	
Don’t know	5 (0.7)	6 (4.8)	5 (1.3)	16 (1.3)	(0–6.8)	
Have you ever had a flu vaccination? (n = 1222)						
Yes	460 (64.2)	50 (40.3)	221 (57.9)	731 (59.8)	(56.2–63.3)	<0.001
No	256 (35.8)	74 (59.7)	161 (42.1)	491 (40.2)	(35.8–44.5)	

*p*-value = calculated for the Chi-square test group. * vaccinated sample.

**Table 4:** Beliefs and occurrence of adverse effects after the administration of the COVID-19 vaccine. (n = 1222).

	Nursing n (%)	Pharmacy n (%)	Medicine n (%)	Total n (%)	IC 95%	*p*-Value
Does it produce more adverse effects than other vaccines? (n = 1204)						
Yes	61 (8.6)	39 (15.4)	16 (9.8)	116 (9.6)	(4.2–15.0)	0.132
No	487 (68.4)	74 (60.2)	239 (64.8)	800 (66.4)	(63.1–69.7)	
Don’t know	164 (23.0)	30 (24.4)	94 (25.5)	288 (23.9)	(18.9–28.8)	
Do there exist unknown adverse effects of the COVID-19 vaccine? (n = 1222)						
Yes	266 (37.2)	59 (47.6)	136 (35.6)	461 (37.7)	(33.3–42.1)	0.026
No	99 (13.8)	23 (18.5)	62 (16.2)	184 (15.1)	(9.9–20.3)	
Don’t know	351 (49.0)	42 (33.9)	184 (48.2)	577 (47.2)	(43.1–51.3)	
Do you trust in the recommendations of the health authorities? (n = 1219)						
Yes	651 (91.2)	101 (82.1)	335 (87.7)	1087 (89.2)	(87.3–91.0)	0.035
No	61 (8.5)	21 (17.1)	45 (11.8)	127 (10.4)	(5.1–15.7)	
Don’t know	2 (0.3)	1 (0.8)	2 (0.5)	5 (0.4)	(0–5.9)	
After vaccination, have you suffered adverse effects? (n = 606)						
Yes	374 (86.4)	22 (91.7)	123 (82.6)	519 (85.6)	(82.6–88.6)	0.298
No	59 (13.6)	2 (8.3)	26 (17.4)	87 (14.4)	(7.0–21.8)	
What adverse effects have you suffered? * n = 374, n = 22, n = 123, n = 519						
Pain at injection site	291 (77.8)	16 (72.7)	84 (68.3)	391 (75.3)	(71.0–79.6)	0.101
Tiredness	205 (54.8)	13 (59.1)	62 (50.4)	280 (53.9)	(48.1–59.7)	0.616
Fever	174 (46.5)	17 (77.3)	62 (50.4)	253 (48.7)	(42.5–54.8)	0.018
Generalized muscle pain	174 (46.5)	11 (50.0)	57 (46.3)	242 (46.6)	(40.3–52.9)	0.948
Headache	186 (49.7)	11 (50.0)	38 (30.9)	235 (45.3)	(38.9–51.6)	0.001
Shivering	140 (37.4)	14 (63.6)	42 (34.1)	196 (37.8)	(31.0–44.6)	0.031
Swollen glands	28 (7.5)	1 (4.5)	14 (11.4)	43 (8.3)	(0.1–15.5)	0.321
Vomiting	22 (5.9)	3 (13.6)	10 (8.1)	35 (6.7)	(0.0–15.0)	0.290
Other	22 (5.9)	1 (4.5)	3 (2.4)	26 (5.0)	(0.0–13.3)	0.314

*p*-value = calculated for the Chi-square test group. * Number of vaccinated grouped by degrees.

**Table 5:** Proportion of the occurrence of adverse effects depending on the type of COVID-19 vaccine received and multivariate logistic regression analysis.

	Total	Vaxzevria^®^	Comirnaty^®^	Spikevax^®^	Chi^2^	Vaxzevria^®^ vs. Comirnaty^®^	*p*-Value	Vaxzevria^®^ vs. Spikevax^®^	*p*-Value
	519 (100)	229 (44.1)	221 (42.6)	69 (13.3)					
Variable	n (%)	n (%)	n (%)	n (%)		OR (95% CI)		OR (95% CI)	
Pain at injection site									
No (*R*)	128 (24.7)	62 (27.1)	52 (23.5)	14 (20.3)	0.454	1	1	1	1
Yes	391 (75.3)	167 (72.9)	169 (76.5)	55 (79.7)		1.59 (0.92–2.73)	0.095	2.40 (1.11–5.18)	0.026
Tiredness									
No (*R*)	239 (46.1)	77 (33.6)	119 (53.8)	43 (62.3)	<0.001	1	1	1	1
Yes	280 (53.9)	152 (66.4)	102 (46.2)	26 (37.7)		1.04 (0.60–1.78)	0.892	0.71 (0.34–1.49)	0.361
Fever									
No (*R*)	266 (51.3)	55 (24.0)	162 (73.3)	49 (71.0)	<0.001	1	1	1	1
Yes	253 (48.7)	174 (76.0)	59 (26.7)	20 (29.0)		0.18 (0.11–0.28)	<0.001	0.27 (0.13–0.54)	<0.001
Generalized muscle pain									
No (*R*)	277 (53.4)	86 (37.6)	144 (65.2)	47 (68.1)	<0.001	1	1	1	1
Yes	242 (46.6)	143 (62.4)	77 (34.8)	22 (31.9)		0.70 (0.42–1.16)	0.168	0.79 (0.39–1.61)	0.513
Headache									
No (*R*)	284 (54.7)	94 (41.0)	143 (64.7)	47 (68.1)	<0.001	1	1	1	1
Yes	235 (45.3)	135 (59.0)	78 (35.3)	22 (31.9)		0.68 (0.41–1.13)	0.140	0.62 (0.31–1.25)	0.183
Shivering									
No (*R*)	323 (62.2)	97 (42.4)	168 (76.0)	58 (84.1)	<0.001	1	1	1	1
Yes	196 (37.8)	132 (57.6)	53 (24.0)	11 (15.9)		0.58 (0.35–0.99)	0.044	0.29 (0.13–0.67)	0.003
Swollen glands									
No (*R*)	476 (91.7)	211 (921)	203 (91.9)	62 (89.9)	0.829	1	1	1	1
Yes	43 (8.3)	18 (7.9)	18 (8.1)	7 (10.1)		2.49 (1.06–5.85)	0.056	3.12 (1.05–9.29)	0.052
Vomiting									
No (*R*)	484 (93.3)	197 (86.0)	219 (99.1)	68 (98.6)	<0.001	1	1	1	1
Yes	35 (6.7)	32 (14.0)	2 (0.9)	1 (1.4)		0.09 (0.02–0.45)	0.003	0.17 (0.02–1.49)	0.110

*p*-value = calculated for the Chi-square test group.

## References

[1] Desmond A., Offit P.A. (2021). On the Shoulders of Giants—From Jenner’s Cowpox to mRNA Covid Vaccines. N. Engl. J. Med..

[2] Marco V. (2020). COVID-19 vaccines: The pandemic will not end overnight. Lancet Microbe.

[3] Andre F.E., Booy R., Bock H.L., Clemens J., Datta S.K., John T.J., Lee B.W., Lolekha S., Peltola H., Ruff T.A. (2008). Vaccination greatly reduces disease, disability, death and inequity worldwide. Bull. World Health Organ..

[4] Reintjes R., Das E., Klemm C., Richardus J.H., Keßler V., Ahmad A. (2016). “Pandemic Public Health Paradox”: Time Series Analysis of the 2009/10 Influenza A/H1N1 Epidemiology, Media Attention, Risk Perception and Public Reactions in 5 European Countries. PLoS ONE.

[5] Thomson A., Vallée-Tourangeau G., Suggs L.S. (2018). Strategies to increase vaccine acceptance and uptake: From behavioral insights to context-specific, culturally-appropriate, evidence-based communications and interventions. Vaccine.

[6] Albrecht D. (2022). Vaccination, politics and COVID-19 impacts. BMC Public Health.

[7] Lazarus J.V., Ratzan S.C., Palayew A., Gostin L.O., Larson H.J., Rabin K., Kimball S., El-Mohandes A. (2021). A global survey of potential acceptance of a COVID-19 vaccine. Nat. Med..

[8] Ali M., Hossain A. (2021). What is the extent of COVID-19 vaccine hesitancy in Bangladesh? A cross-sectional rapid national survey. BMJ Open.

[9] Secosan I., Virga D., Crainiceanu Z., Bratu L., Bratu T. (2020). Infodemia: Another Enemy for Romanian Frontline Healthcare Workers to Fight during the COVID-19 Outbreak. Medicina.

[10] Gagneur A., Gosselin V., Dubé È. (2018). Motivational interviewing: A promising tool to address vaccine hesitancy. Vaccine.

[11] Farooq F., Rathore F.A. (2021). COVID-19 Vaccination and the Challenge of Infodemic and Disinformation. J. Korean Med. Sci..

[12] Pérez Milena A. (2021). El desafío de informar a la población como estrategia para la cobertura óptima de la vacunación contra la COVID-19. Comunidad.

[13] Agencia Española del Medicamento y Productos Sanitarios (2021). Vigilancia de la Seguridad de las Vacunas Frente a la COVID-19. [Internet] Madrid: Ministerio de Sanidad. https://www.aemps.gob.es/medicamentosUsoHumano/vacunas/docs/vigilancia_seguridad_vacunas_COVID-19.pdf?.

[14] Petousis-Harris H. (2020). Assessing the Safety of COVID-19 Vaccines: A Primer. Drug Saf..

[15] Matić Z., Šantak M. (2021). Current view on novel vaccine technologies to combat human infectious diseases. Appl. Microbiol. Biotechnol..

[16] Azimi M., Dehzad W.M., Atiq M.A., Bahain B., Asady A. (2021). Adverse Effects of the COVID-19 Vaccine Reported by Lecturers and Staff of Kabul University of Medical Sciences, Kabul, Afghanistan. Infect. Drug Resist..

[17] Jeon M., Kim J., Oh C.E., Lee J.-Y. (2021). Adverse Events Following Immunization Associated with Coronavirus Disease 2019 Vaccination Reported in the Mobile Vaccine Adverse Events Reporting System. J. Korean Med. Sci..

[18] Working Group on Vaccine Hesitancy (SAGE) Report of the SAGE Working Group on Vaccine Hesitancy. https://www.who.int/immunization/sage/meetings/2014/october/1_Report_WORKING_GROUP_vaccine_hesitancy_final.pdf.

[19] World Health Organization (WHO) Vaccination: European Commission and World Health Organization Join Forces to Promote the Benefits of Vaccines. https://www.who.int/news/item/12-09-2019-vaccination-european-commission-and-world-health-organization-join-forces-to-promote-the-benefits-of-vaccines.

[20] Freed G.L., Clark S.J., Butchart A.T., Singer D.C., Davis M.M. (2011). Sources and Perceived Credibility of Vaccine-Safety Information for Parents. Pediatrics.

[21] Vorsters A., Tack S., Hendrickx G., Vladimirova N., Bonanni P., Pistol A., Metličar T., Pasquin M.A., Mayer M., Aronsson B. (2010). A summer school on vaccinology: Responding to identified gaps in pre-service immunisation training of future health care workers. Vaccine.

[22] Siddiqui M., Salmon D.A., Omer S.B. (2013). Epidemiology of vaccine hesitancy in the United States. Hum. Vaccines Immunother..

[23] Kernéis S., Jacquet C., Bannay A., May T., Launay O., Verger P., Pulcini C., Abgueguen P., Ansart S., Bani-Sadr F. (2017). Vaccine Education of Medical Students: A Nationwide Cross-sectional Survey. Am. J. Prev. Med..

[24] Barello S., Nania T., Dellafiore F., Graffigna G., Caruso R. (2020). ‘Vaccine hesitancy’ among university students in Italy during the COVID-19 pandemic. Eur. J. Epidemiol..

[25] Dror A.A., Eisenbach N., Taiber S., Morozov N.G., Mizrachi M., Zigron A., Srouji S., Sela E. (2020). Vaccine hesitancy: The next challenge in the fight against COVID-19. Eur. J. Epidemiol..

[26] Gómez Marco J.J., Álvarez Pasquín M.J., Martín Martín S. (2021). Efectividad y seguridad de las vacunas para el SARS-CoV-2 actualmente disponibles. FMC.

[27] Grupo de Trabajo Técnico de Vacunación COVID-19 de la Ponencia de Programa y Registro de Vacunaciones. Información Sobre las Vacunas en Tiempos de COVID-19. Consejo Interterritorial del SNS. Ministerio de Sanidad. https://www.mscbs.gob.es/profesionales/saludPublica/prevPromocion/vacunaciones/covid19/.

[28] Olmedo Lucerón C., Limia Sánchez A., García Gómez M. (2021). La vacunación frente a la COVID-19 en colectivos laborales. Rev. Esp. Salud Pública.

[29] Sharma M., Davis R., Wilkerson A. (2021). COVID-19 Vaccine Acceptance among College Students: A Theory-Based Analysis. Int. J. Environ. Res. Public Health.

[30] Tavolacci M., Dechelotte P., Ladner J. (2021). COVID-19 Vaccine Acceptance, Hesitancy, and Resistancy among University Students in France. Vaccines.

[31] Mo P., Luo S., Wang S., Zhao J., Zhang G., Li L., Li L., Xie L., Lau J. (2021). Intention to Receive the COVID-19 Vaccination in China: Application of the Diffusion of Innovations Theory and the Moderating Role of Openness to Experience. Vaccines.

[32] Grech V., Gauci C. (2020). WITHDRAWN: Vaccine hesitancy in the University of Malta Faculties of Health Sciences, Dentistry and Medicine vis-à-vis influenza and novel COVID-19 vaccination. Early Hum. Dev..

[33] Karafillakis E., Dinca I., Apfel F., Cecconi S., Wűrz A., Takacs J., Suk J., Celentano L.P., Kramarz P., Larson H.J. (2016). Vaccine hesitancy among healthcare workers in Europe: A qualitative study. Vaccine.

[34] Qiao S., Tam C.C., Li X. (2021). Risk Exposures, Risk Perceptions, Negative Attitudes Toward General Vaccination, and COVID-19 Vaccine Acceptance Among College Students in South Carolina. Am. J. Health Promot..

[35] Qiao S., Friedman D.B., Tam C.C., Zeng C., Li X. (2020). Vaccine acceptance among college students in South Carolina: Do information sources and trust in information make a difference?. medRxiv.

[36] Saied S.M., Saied E.M., Kabbash I.A., Abdo S.A.E. (2021). Vaccine hesitancy: Beliefs and barriers associated with COVID-19 vaccination among Egyptian medical students. J. Med. Virol..

[37] Gallè F., Sabella E.A., Roma P., De Giglio O., Caggiano G., Tafuri S., Da Molin G., Ferracuti S., Montagna M.T., Liguori G. (2021). Knowledge and Acceptance of COVID-19 Vaccination among Undergraduate Students from Central and Southern Italy. Vaccines.

[38] Sallam M., Dababseh D., Eid H., Hasan H., Taim D., Al-Mahzoum K., Al-Haidar A., Yaseen A., Ababneh N., Assaf A. (2021). Low COVID-19 Vaccine Acceptance Is Correlated with Conspiracy Beliefs among University Students in Jordan. Int. J. Environ. Res. Public Health.

[39] Pastorino R., Villani L., Mariani M., Ricciardi W., Graffigna G., Boccia S. (2021). Impact of COVID-19 Pandemic on Flu and COVID-19 Vaccination Intentions among University Students. Vaccines.

[40] Sallam M. (2021). COVID-19 Vaccine Hesitancy Worldwide: A Concise Systematic Review of Vaccine Acceptance Rates. Vaccines.

[41] Lambert P.-H., Podda A. (2018). Education in Vaccinology: An Important Tool for Strengthening Global Health. Front. Immunol..

[42] Kelekar A.K., Lucia V.C., Afonso N.M., Mascarenhas A.K. (2021). COVID-19 vaccine acceptance and hesitancy among dental and medical students. J. Am. Dent. Assoc..

[43] Le An P., Nguyen H.T.N., Nguyen D.D., Vo L.Y., Huynh G. (2021). The intention to get a COVID-19 vaccine among the students of health science in Vietnam. Hum. Vaccines Immunother..

[44] Wagner A.L., Masters N.B., Domek G.J., Mathew J.L., Sun X., Asturias E.J., Ren J., Huang Z., Contreras-Roldan I.L., Gebremeskel B. (2019). Comparisons of Vaccine Hesitancy across Five Low- and Middle-Income Countries. Vaccines.

[45] Lucia V.C., Kelekar A., Afonso N.M. (2020). COVID-19 vaccine hesitancy among medical students. J. Public Health.

[46] Szmyd B., Bartoszek A., Karuga F.F., Staniecka K., Błaszczyk M., Radek M. (2021). Medical Students and SARS-CoV-2 Vaccination: Attitude and Behaviors. Vaccines.

[47] Rodríguez-Blanco N., Montero-Navarro S., Botella-Rico J., Felipe-Gómez A., Sánchez-Más J., Tuells J. (2021). Willingness to Be Vaccinated against COVID-19 in Spain before the Start of Vaccination: A Cross-Sectional Study. Int. J. Environ. Res. Public Health.

[48] Gagneux-Brunon A., Detoc M., Bruel S., Tardy B., Rozaire O., Frappe P., Botelho-Nevers E. (2021). Intention to get vaccinations against COVID-19 in French healthcare workers during the first pandemic wave: A cross-sectional survey. J. Hosp. Infect..

[49] Nguyen K., Srivastav A., Razzaghi H., Williams W., Lindley M.C., Jorgensen C., Abad N., Singleton J.A. (2021). COVID-19 vaccination intent, perceptions, and reasons for not vaccinating among groups prioritized for early vaccination—United States, September and December 2020. Am. J. Transplant..

[50] Mascarenhas A.K., Lucia V.C., Kelekar A., Afonso N.M. (2021). Dental students’ attitudes and hesitancy toward COVID-19 vaccine. J. Dent. Educ..

[51] Van Der Weerd W., Timmermans D.R., Beaujean D.J., Oudhoff J., Van Steenbergen J.E. (2011). Monitoring the level of government trust, risk perception and intention of the general public to adopt protective measures during the influenza A (H1N1) pandemic in the Netherlands. BMC Public Health.

